# Does Climate Play Any Role in COVID-19 Spreading?—An Australian Perspective

**DOI:** 10.3390/ijerph18179086

**Published:** 2021-08-28

**Authors:** Joji Abraham, Christopher Turville, Kim Dowling, Singarayer Florentine

**Affiliations:** 1 School of Engineering, Information Technology and Physical Sciences, Federation University Australia, Mt Helen Campus, Ballarat, VIC 3353, Australia; c.turville@federation.edu.au (C.T.); k.dowling@federation.edu.au (K.D.); 2 Department of Geology, University of Johannesburg, Johannesburg 2006, South Africa; 3 Future Regions Research Centre, School of Science, Psychology and Sport, Federation University Australia, Mt Helen Campus, Ballarat, VIC 3353, Australia; S.florentine@federation.edu.au

**Keywords:** coronavirus disease, climate and COVID-19, SARS-CoV-2, solar radiation and COVID-19, ultraviolet index, weather factors and COVID-19

## Abstract

Compared to other countries, the COVID-19 pandemic did not severely affect Australia as measured by total deaths until mid-2021. Though a substantial number of daily confirmed cases (up to 698) were reported during the second wave, most of them were from the southern state of Victoria. This study examined the possible correlations between climate variables and the number of daily confirmed COVID-19 cases in Victoria, Australia, from 25 January to 31 October 2020. Appropriate regression models and cross-correlation diagnostics were used to examine the effect of temperature, rainfall, solar exposure, and ultraviolet index (UVI) with the number of daily confirmed cases. Significant positive associations were identified for solar exposure and maximum and average UVI for confirmed cases one and 19 days later. Negative associations for these variables were found for confirmed cases five days later. Minimum temperature had a significant negative correlation one day later and a positive effect 21 days later. No significant correlation was found for maximum temperature and rainfall. The most significant relationships were found for confirmed cases 19 days after changes in the meteorological variables. A 1% increase in solar exposure, maximum UVI, and average UVI was associated with a 0.31% (95% CI: 0.13 to 0.51), 0.71% (95% CI: 0.43 to 0.98), and 0.63% (95%CI: 0.20 to 1.61) increase 19 days later in the number of confirmed cases, respectively. The implications of these results can be used in the public health management of any possible future events in Australia. It also highlights the significance of considering the climatic variables and seasonality in all kinds of epidemics and pandemics.

## 1. Introduction

As of the end of 2020, no country (excluding small island nations) is free of the COVID-19 virus. Indeed, the pandemic returned as a second wave in many European countries and the USA and appeared as the first wave in several Asian and African countries. Outbreaks have seriously challenged governments across the world, and it has thrown enormous pressure on their health systems. As of 23 June 2021, Australia conducted 19,934,238 COVID-19 tests and identified 30,378 cases only (0.2%), but with 910 fatalities [1]. All federal, states, territories, and local governments worked with residents to manage the COVID-19 pandemic. In 2020, the Australian second wave was generally not significant, with the exception of the states of Victoria and New South Wales (NSW). In Victoria, the second wave began in mid-June 2020, with the maximum peak observed in the first week of August 2020. However, after considerable intervention strategies, including restriction of movement, by the end of October, case numbers were very low and largely linked to returning overseas travellers. Significant developments have occurred in NSW since the middle of 2021 and as of the end of August 2021. There were more than 10,000 active cases largely in NSW and Victoria, which represent the southernmost mainland states that also have the highest population centres with the Australian human population at 25.8 million, of which 25% live in Victoria [1].

Approximately two months after the emergence of COVID-19, a debate arose regarding the influence of climate factors in SARS-CoV-2 transmission. Particular interest was focused on quick transmission and high mortality rates in the high northern latitudes, specifically in Europe and the USA, compared to countries in the equatorial regions. Interest in the role of exposure to solar radiation and vitamin-D levels as important climate-related variables quickly emerged. In this respect, the rapid spread of COVID-19 in colder countries is relatively similar to that of influenza dispersion in that it spreads aggressively in winter and retreats in summer [2,3]. In European countries and the USA, influenza spreads in the northern winter from December to March, while in Australia, it happens from May to July (southern winter). Because of the apparent link to seasonality, it appears that an important question to investigate is whether climate factors modulate the progression of COVID-19. This is crucial for epidemiologists, healthcare decision-makers, and governments at various levels since it will directly and critically inform their management plans [4]. It is already known that a major transmission route for the virus is respiratory droplets passing through the atmosphere, and it has been reported that the mobility of coughing droplets from an infected person depends on atmospheric parameters, such as humidity, ambient air temperature, and wind speed [5].

Studies conducted across many regions suggest links between climate factors and COVID-19 transmission and mortality and, dependent on the variable, either a direct or inverse relationship [4,6,7]. For example, Takagi et al. [8] observed that temperature, exposure time to solar radiation, and ultraviolet index (UVI) are negatively related to COVID-19 transmission, whereas wind speed and increasing sky cover are positively related.

Prior to the publications of other related studies, Rhodes et al. [9] note that latitude (the location of the country) is a significant factor in determining the extent of COVID-19 spreading and mortality, observing that there was a 4.9% increase in mortality for each degree of latitude movement towards the north. In support of this view, and analysing data gathered from 208 territories, Quilodran et al. [4] reported that (i) each degree increase in atmospheric temperature decreased the mortality rate by ~4%, (ii) a 1% increase in relative humidity raised the mortality rate by ~2%, while (iii) a unit increase in UVI reduced the mortality rate by ~15%. Similarly, Ramirez and Lee [6] analysed the UVI and humidity data of 205 cities in 21 countries and found a direct association with humidity but a negative association of COVID-19 spread with UVI. A study from Brazil highlighted a similar association between weather factors (temperature, UVI, ozone concentration, and cloud cover) with COVID-19 growth rate and daily deaths; specifically, a unit increase in UVI stimulated a 6% decline in the mortality rate [7]. A similar pattern was also identified by Islam et al. [10] after analysing data from 310 regions worldwide. In contrast, Yao et al. [11] found no association between COVID-19 spread with temperature in the Wuhan area in China.

UV exposure is linked to increased rates of skin cancer development [12]. However, it is also known that UV has antimicrobial properties and is commonly used in several healthcare and water treatment facilities [13]. It also has a significant association with vitamin-D formation [14], and other studies have shown direct UV effects on the human immune system [15]. Particulate matter (PM) in the atmosphere has also been associated directly with the rate of change in the daily number of COVID-19 cases [16]. In addition to climate factors, it is clear that physical factors also contribute to virus spreading and mortality rates, such as human comorbidities, physical contact frequencies, innate immunity levels, appropriate use of personal protection equipment (PPE) and personal hygiene practices, the presence of various types of fomites, the structure of lock-down measures, and the features of available health systems [17,18,19].

Although there have been some valuable studies published, the association between climate factors and COVID-19 transmission has not been closely investigated, particularly with regard to the Australian context. Since most of Australia’s COVID-19 cases have been reported from the states of Victoria and NSW (especially the metropolitan areas), the objective of this study is to understand the influence of climate factors in spreading the COVID-19 using Melbourne Australia as a case study. Most of the published studies have used first wave data only [20,21,22], but this study considers both the first and second wave data available for this location.

## 2. Methodology

### 2.1. Data Collection

The COVID-19 daily new case and UVI index data from Victoria between 25 January and 31 October 2020 were collected from the Victorian Health Department website [23] and the Australian Radiation Protection and Nuclear Safety Agency (ARPANSA), respectively. Other necessary weather data such as temperature, humidity, and rainfall were collected from the Bureau of Meteorology (BOM), Australia [24]. The starting and ending date was selected to match the initial confirmed cases of COVID-19 in Victoria until no more confirmed cases were diagnosed for more than a month.

### 2.2. Statistical Analysis

Most of the meteorological variables examined in this study were highly correlated, so their effects on COVID-19 were examined separately to remove the errors due to collinearity. Initial correlations were determined for each of the variables with confirmed cases. These correlations may be spurious as problems of non-homogeneity of variance, non-stationarity, and autocorrelated time series were identified. Each time series was log-transformed to stabilise the variance (adding 1 to remove the problem of taking the log of 0). The first differences of the logs of each time series were then taken to eliminate any trends present. The augmented Dicky Fuller test confirmed that each time series was stationary due to this process.

Cross-correlations that compare all the investigated variables with the number of confirmed cases were used to identify significant correlations. It is well known that autocorrelation within a time series can produce spurious cross-correlations [25]. Pre-whitening was used to remove the autocorrelations from the number of confirmed cases and applied the same filter to each climate variable. This process enables a more meaningful investigation of the cross-correlation functions (CCFs) to identify predictive relationships. Various authors have previously used pre-whitening to limit the effect of autocorrelation when examining climatological and environmental time series [26,27,28,29].

The pre-whitening process used in this study initially examined the number of confirmed cases. Several auto-regressive integrated moving average (ARIMA) models, with appropriate parameters determined from the inspection of the autocorrelated functions (ACF) and partial autocorrelation functions (PACF) plots, were attempted. The selection of ARIMA models to estimate confirmed cases of COVID-19 was based on the examination of the time series and the success of various other studies that utilised ARIMA models for pandemics. ARIMA models have been conducted on previous pandemics such as severe acute respiratory syndrome (SARS) [30] and the incidence of influenza in China [31]. ARIMA models have also been applied to COVID-19, and a summary of these can be found in Lee et al. [32]. The most appropriate ARIMA model was determined using Akaike’s information criteria (AIC) [33]. The same model was then applied to each of the meteorological variables. The CCF of the residuals from the confirmed cases and the filtered values (defined as the difference between the observed and model estimates) from the meteorological variable factor was then examined to identify any significant lagged correlations. This process identified the number of days required to change the meteorological variable to significantly impact the number of confirmed cases.

A linear regression model was constructed for each meteorological variable from all of the significant lags identified from the CCF against the number of confirmed cases. This allowed the identification of significant lags when all data was considered together. For all tests conducted, *p* < 0.05 was considered statistically significant, and all analyses were performed using R statistical software (version 4.0.3) (R Foundation for Statistical Computing, Vienna, Austria).

## 3. Results

**Figure 1:**
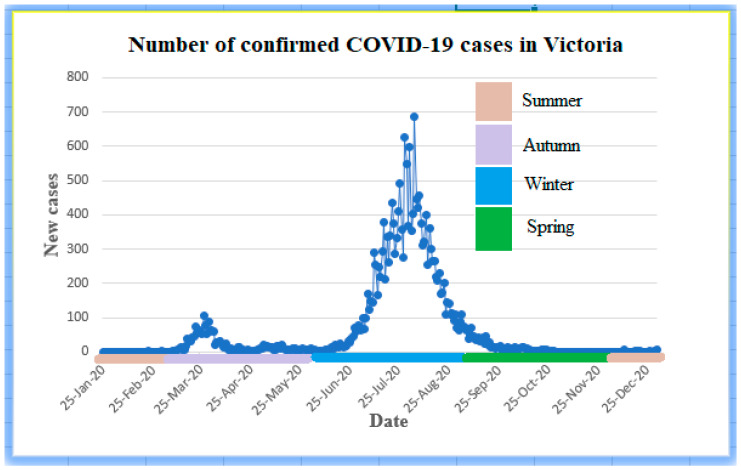
An association was observed between season and daily new COVID-19 cases. The second wave that occurred in winter has a high peak compared to the first wave.

Spatial and temporal heterogeneities occur in association with COVID-19 spreading and with meteorological parameters like ambient air temperature, humidity, and UVI. While plotting the daily case data, two wave patterns are observed. The first case of COVID-19 reported in Victoria was on 25 January 2020, and the highest daily case reported during the first wave was on 27 March 2020, followed by a decline in case numbers [23]. The second wave began to rise in the second week of June 2020, and the wave reached its peak on 4th August and started to decline [23]. The first wave was found to be smaller when compared to the second wave, a pattern that is noted in many jurisdictions. A contributing mechanism behind this variation may be the atmospheric temperature and UVI index because the first wave started during the summer and peaked in the autumn. In contrast, the second wave occurred during the winter (Figure 1), where temperature and UVI were comparatively low. The influence of season and climate parameters, precisely atmospheric temperature, and humidity, on many viruses, were mentioned in several previous studies [2,3,34,35].

Similar to the above, many studies showed an inverse association between air temperature and COVID-19 spreading [36,37,38,39,40], with a significant correlation between recovery time and solar radiation exposure [41]. Solar radiation, specifically UV radiation, has the ability to inactivate the virus and boost the immune system through vitamin-D preparation and thereby slow the infection rate [42,43,44,45]

### 3.1. Descriptive Analyses

Table 1 shows the descriptive statistics for the daily confirmed cases and meteorological variables. There were 20,269 confirmed new cases over the time period examined, and these showed a daily average of 72.39, with the highest day observation being 687 cases. The minimum solar exposure was 2.1, with a maximum of 30.1. The minimum and maximum values of maximum UVI were 0.3 and 10.8, respectively, and that of average UVI was 0.4 and 3, respectively. The minimum and maximum values of minimum temperatures were 0.2 °C and 23 °C, respectively, whereas maximum temperatures were 14.3 °C and 43.6 °C, respectively. The rainfall in the data collection period was low, with an average of 1.91 mm (minimum 0 and maximum 69 mm) recorded.

### 3.2. Correlation Analysis of Original and Stationary Times Series

**Figure 2:**
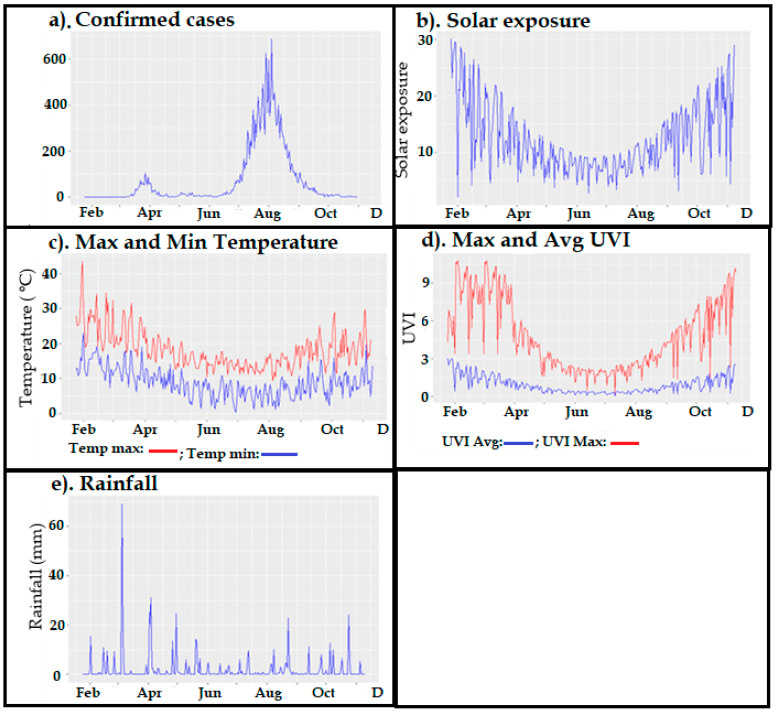
Original time series of the daily confirmed cases (**a**) and six meteorological variables (**b**) solar exposure; (**c**) maximum and minimum temperature; (**d**) maximum and average UVI; (**e**) rainfall.

Seven-time series that depict the confirmed cases and climate variables are shown in Figure 2. It is clear that there are trends and non-homogeneity of variance present for all of the time series; that is, all of these time series are not stationary (in terms of mean and variance at all time points). Each of these time series was log-transformed prior to the first differences being taken to make them stationary. This assured that any correlations would be without trend confounders. The augmented Dicky Fuller test confirmed that each time series was stationary as a result of this process.

The Pearson and Spearman correlation coefficients of the original and stationary time series are shown in Table 2. All meteorological variables, excluding rainfall, were significant for the original time series. However, only the length of solar exposure and max UVI were significant once the trend had been removed, indicating that the trends made the original correlations appear larger. The similarity in results between the two types of correlations, together with the fact that the residuals were reasonably normally distributed, meant that the Pearson correlation was deemed appropriate to use for the remainder of the study.

**Figure 3:**
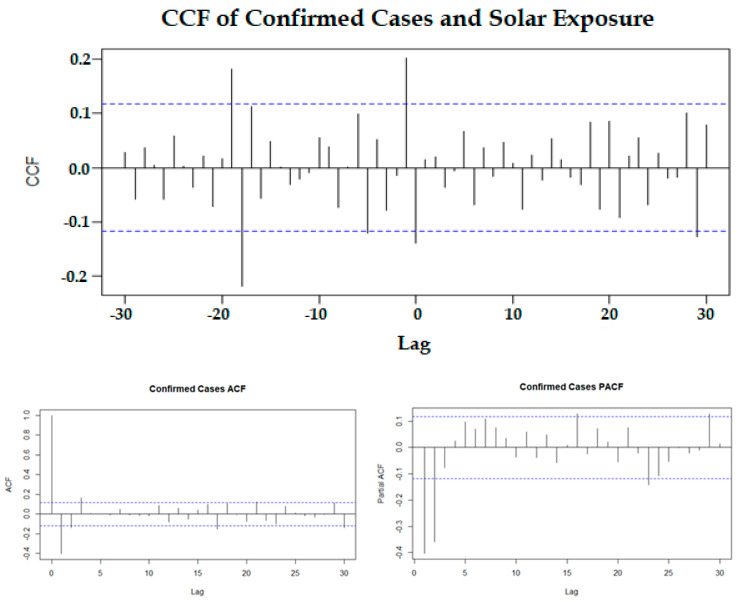
Cross-correlation function (CCF) between confirmed cases and solar exposure (the negative lags indicate that solar exposure is before the confirmed cases) (**top**). Autocorrelation function (ACF) and partial autocorrelation function (PACF) for the stationary confirmed cases (**bottom**).

The correlations from Table 2 indicate that solar exposure and max UVI affected the confirmed cases. These correlations are determined by assuming that the effect of the meteorological variable and the onset of confirmed cases were on the same day (identified as lag 0). However, it is very possible that the effects of the meteorological variables could impact the number of confirmed cases in future observations. The cross-correlation function (CCF) is used to determine the correlations of confirmed cases with each climate variable with a lag of up to 30 days. Figure 3 shows the CCF with the correlations between the confirmed cases and solar exposure. The CCF indicates that there are significant correlations (beyond the blue dashed line) at the lag time(s) of 0, 1, 5, 18, and 19 days before the case confirmation. The effect on confirmed cases is positive after lags of 1 and 19 days and negative after lags of 0, 5, and 18 days. That is, if the solar exposure increases on one day, then there are increases in cases for 1 and 19 days later and a decrease in cases on that day as well as 5 and 18 days later. Significant lag times are those below 0, which indicates that solar exposure precedes and influences the number of cases. The CCF shows that there are significant correlations (beyond the blue dashed line) at the lag time(s) of 0, 1, 5, 18, and 19 days before the case confirmation.

The cross-correlations could be conducted as above on all of the stationary series. However, these correlations may still be misleading as there could be spurious correlations present based on temporal dependencies between the adjacent values of a time series (autocorrelations). Pre-whitening removes these dependencies and allows a better investigation of the CCF [28,29]. That is, pre-whitening identifies the linear association between two time series from their autocorrelation. The following section discusses the pre-whitening process and the correlations identified after this process. 

### 3.3. Correlation Analysis after Pre-Whitening the Time Series

The process of pre-whitening initially selected an appropriate ARIMA model for the stationary confirmed cases time series in order to appropriately fit the data. The model was then subsequently applied to each of the environmental factors. The CCF of the residuals from the confirmed cases and the filtered values (the difference between the observed and model estimates) from the environmental factors were then examined to identify any significant lagged correlations.

**Figure 4:**
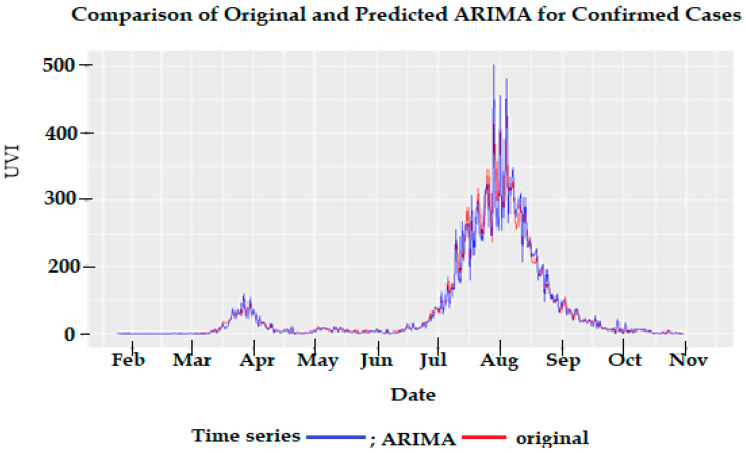
Comparison of the confirmed number of cases with the predictions from the auto-regressive integrated moving average (2,1,2) model.

Figure 3 (bottom) shows the ACF and PACF plots of the confirmed cases. These plots indicate that between an order of 2–3 should be investigated for both the moving average (MA) and autoregressive (AR) components of the ARIMA model. Several ARIMA models of the order 2–3 were fitted to the data, and after extensive modelling, the optimum model was selected to address the AIC, the significance of its parameters, and considerations of parsimony. The best model selected was an ARIMA (2,1,2) model indicating that an order of 2 was appropriate for both the autoregressive and moving average components. The results of the final ARIMA model are shown in Table 3. The performance of the predicted values from ARIMA (2,1,2) to the original number of cases is shown in Figure 4. The model performs reasonably well except when there are large daily changes in the original number of cases. The ACF of residuals from this model indicates that there is no more autocorrelation in the series.

**Figure 5:**
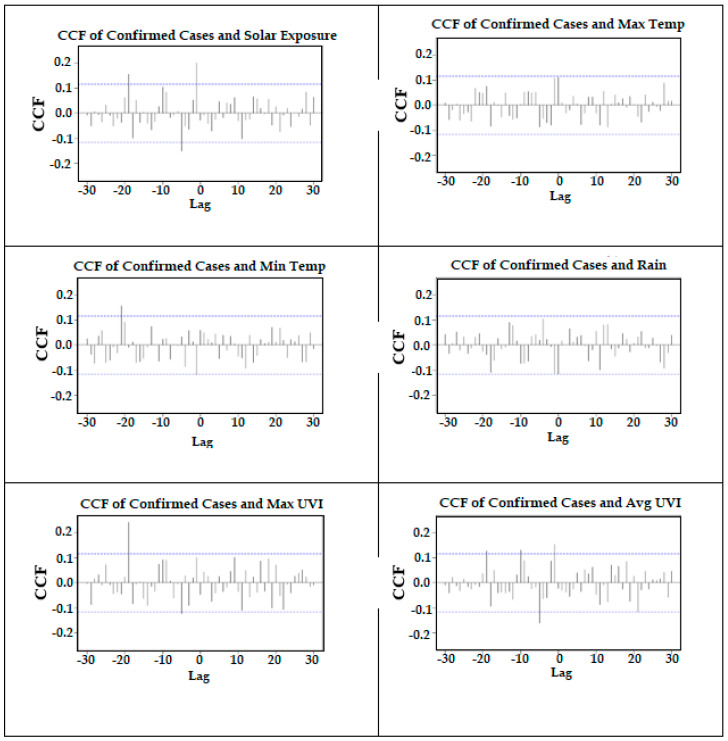
CCF of confirmed cases against each meteorological variable.

The next step of the pre-whitening stage involved filtering the meteorological variables by finding the difference between their original values (of the stationary series) and the predicted values using the ARIMA (2,1,2) model. Figure 5 shows the CCFs of the residuals of the ARIMA (2,1,2) for confirmed cases against the filtered values for each environmental factor. Note that it is the correlations on the left side of the Figure that relate to the instances in which the climate variable occurs before the confirmed cases. The significant correlations identified for each of the meteorological variables from Figure 5 are summarised in Table 4. Rainfall did not have any significant correlations with confirmed cases. Of particular interest is that while the maximum temperature was not significant, minimum temperatures had significant correlations at a lag of 1 and 21 days.

### 3.4. Regression on Significant Correlations

As a final check of the importance of the lagged correlation, linear regression analyses on the confirmed cases against each climate variable were conducted with all the significant lags identified in the previous step. The regression analyses also identified any lags that were not significant when considered together with other results and provided useful coefficients to describe the relationship between lag time and confirmed cases. Table 5 shows the results of the significant lags from these regression analyses. Only the average UVI lag 10 was found to be non-significant when incorporated with other results. The regression coefficients from Table 5 can be interpreted as the percentage change in the confirmed cases for every 1% change in the meteorological variable. That is, an increase in 1% to the solar exposure will increase the confirmed cases by 0.32% one day later and 0.31% 19 days later.

**Figure 6:**
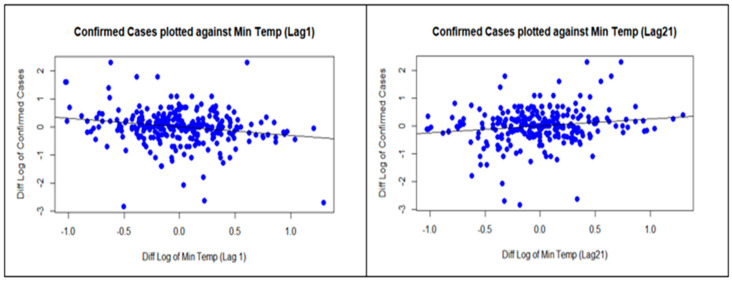
Scatterplots of confirmed cases and minimum temperature at Lag 1 and 21 days.

Solar exposure and the UVI variables showed quite similar patterns: positive correlations at lags of 1 and 19 days and negative correlations at a lag of 5 days. Maximum UVI did not have a significant correlation at lag 1, but inspection of Figure 5 shows that it is marginally non-significant. The minimum temperature has a significant negative correlation at a lag of 1 day and a positive correlation at a lag of 21 days. One example is provided to demonstrate how to visually identify the patterns from the regression analyses in Figure 6 using scatterplots of the minimum temperature and confirmed cases at both lags 1 and 21. Large drops in the minimum temperature are associated with large increases in confirmed cases 1 day later. Generally, decreases in the minimum temperature are then associated with decreases in the number of confirmed cases 21 days later.

## 4. Discussion

It has already been reported that the behaviour of micro-organisms in the environment depends on environmental factors, such as ambient air temperature and humidity [46]. Though several studies reported the association between weather factors and COVID-19 spreading, after general straightforward use of either Pearson or Spearman simple correlation, this study used appropriate regression models and cross-correlation diagnostics and removed the errors formed due to collinearity. This study investigated the association of weather factors with COVID-19 spreading in the Melbourne Metropolitan area in Victoria (between Jan and Oct 2020) and found a positive association between solar exposure, maximum and average UVI with COVID-19 spreading, 1 and 19 days later, and a negative association with these parameters five days later. A negative association was found between the minimum temperature and COVID-19 one day later and a positive association 21 days later. A similar study was conducted in Indonesia, a country north of Australia, and found a correlation between average temperature and COVID-19 [22]. A study in Rio de Janeiro, in Brazil, the third most COVID-19 reported country, also observed a strong negative association between solar radiation and maximum and average temperature [47]. Among the weather parameters, temperature (maximum and average) is the most studied. In this study, a positive association between the maximum and average UVI and solar exposure was also observed besides the minimum temperature.

The current study and many related studies suggest that an increase in solar radiation and the subsequent increase in ambient air temperature, specifically the minimum temperature increase, may reduce the COVID-19 infections in humans. Mechanisms are complex; however, two postulations are considered; (i) the increase in solar radiation, specifically the UV radiation, may decrease the SARS-CoV-2 life expectancy by damaging the virus’ lipid layer. This lipid layer damage may reduce the stability, infection potential and may cause inactivation of the virus [48]; (ii) the increase in UV radiation may increase the vitamin-D levels in people that can enhance the immunity, which helps to prevent the viral spreading and the associated symptoms in the body [43,49,50]. The effects of simulated solar radiation (mid-day representing the summer solstice at 40° N) on SARS-CoV-2 suspended in simulated saliva or culture medium was studied by Ratnesar-Shumate et al. [51] and found that the simulated solar radiation rapidly inactivated the virus (90% within 6.8 min in the simulated saliva and 14.3 min in the culture media). This study highlights that the potential for fomite SARS-CoV-2 transmission in the outside environment will be significantly reduced with increased solar radiation. Similar effects can be expected in the air environment, but it may depend on many factors, specifically the humidity.

The negative association of maximum and average UVI on COVID-19 after five days is expected as it takes around five days to self-identify symptoms of the disease (and get tested), but the positive association after one and 19 days, including the solar radiation effects, needs to be further explored. Similar statistical studies are required with data from other areas in Australia (with previous and subsequent outbreaks) and from other countries across the world to check whether a similar association exists or not. Apart from this, further studies are warranted to find the reason behind the positive and negative association between the minimum temperature on COVID-19 one day and 21 days later exposure.

## 5. Conclusions

Through many studies, it is observed that weather factors play a significant role in microbial spreading, specifically in COVID-19. Australia is a large country with significant climatic and weather variations. This study investigated the role of weather factors in spreading the COVID-19 in the Victoria state capital (Melbourne), which reported the highest COVID-19 cases during the second outbreak. Among the studied weather factors (such as solar exposure, maximum and average UVI, minimum and maximum temperature, and rainfall), maximum and average UVI and solar exposure revealed a positive association with COVID-19 after one and 19 days, whereas the same factors revealed a negative association after five days. The minimum temperature revealed a negative association after a day, whereas it showed a positive association after 21 days. The negative influence of average and maximum UVI after five days is considered the required time for the SARS-CoV-2 to multiply and show its presence through the RTPCR test. Other relations, both positive and negative after 1, 19, and 21 days need to be explored further, as do any possible impacts of COVID-19 variants and society or government-imposed restrictions on behaviors. If outside activity, often described as blue-sky exercise, has an impact on viral spread, this must be factored into any future management strategy. 

## Figures and Tables

**Table 1:** Descriptive statistics of the daily confirmed cases and six meteorological variables for the sample period 25 January to 31 October 2020.

Variables	Mean	SD	Min	Max
Confirmed cases	72.39	128.25	0	687
Solar exposure (MJ/m^2^)	12.16	5.88	2.1	30.1
Maximum UVI	4.64	2.71	0.3	10.8
Average UVI	0.89	0.64	0.40	3.03
Maximum Temperature (°C)	18.16	5.46	14.3	43.6
Minimum Temperature (°C)	8.75	4.12	0.2	23.0
Rainfall (mm)	1.91	5.96	0	69

**Table 2:** Pearson and Spearman correlation coefficients between the daily confirmed cases and six meteorological variables for the original and stationary time series with the corresponding *p*-value.

Meteorological Variable	Original Time Series		Stationary Time Series	
	Pearson	Spearman	Pearson	Spearman
Solar Exposure (MJ/m^2^)	−0.265 (<0.001)	−0.349 (<0.001)	−0.139 (0.020)	−0.131 (0.028)
Max UVI	−0.374 (<0.001)	−0.472 (<0.001)	−0.131 (0.029)	−0.172 (0.004)
Average UVI	−0.357 (<0.001)	−0.506 (<0.001)	−0.104 (0.082)	−0.082 (0.170)
Max Temp (°C)	−0.379 (<0.001)	−0.490 (<0.001)	0.076 (0.203)	0.056 (0.355)
Min Temp (°C)	−0.388 (<0.001)	−0.464 (<0.001)	0.112 (0.062)	0.032 (0.595)
Rainfall (mm)	−0.062 (0.299)	−0.010 (0.861)	−0.071 (0.238)	−0.126 (0.035)

**Table 3:** Optimal ARIMA (2,1,2) model for stationary confirmed cases.

	ar1	ar2	ma1	ma2
Coefficient	0.717	−0.199	−1.339	0.646
SE	0.159	0.089	0.144	0.094
*p*-value	<0.001	0.024	<0.001	<0.001
AIC	447.49			

**Table 4:** Significant correlations for each meteorological variable were identified from the CCF after pre-whitening.

Meteorological Parameters	Sig. Lags from CCF	Correlation of Sig. Lags	*p*-Value of Sig. Lags
Solar exposure (MJ/m^2^)	1	0.205	<0.001
	5	−0.155	0.010
	19	0.166	0.007
Max UV	5	−0.126	0.038
	19	0.267	<0.001
Average UV	1	0.158	0.008
	5	−0.165	0.006
	10	0.139	0.023
	19	0.141	0.023
Max Temp (°C)	Nil		
Min Temp (°C)	1	−0.119	0.046
	21	0.162	0.009
Rainfall (mm)	Nil		

**Table 5:** Results from regression analyses on the significant lags identified from the pre-whitening of each meteorological variable.

Meteorological Parameters	Sig. Lags from Regr	Regr Coeff	95% CI	*p*-Value	R-sq
Solar exposure (MJ/m^2^)	1	0.320	0.138, 0.502	<0.001	10.9%
	5	−0.269	−0.457, −0.081	0.004	
	19	0.313	0.133, 0.505	<0.001	
Max Temp (°C)	Nil				
Min Temp(°C)	1	−0.303	−0.493, −0.113	0.002	6.3%
	21	0.238	0.044, 0.432	0.015	
Rainfall (mm)	Nil				
Max UV	5	−0.258	−0.512, 0.004	0.044	10.7%
	19	0.706	0.434, 0.978	<0.001	
Average UV	1	0.468	0.042, 0.894	0.029	7.1%
	5	−0.531	−0.957, −0.105	0.013	
	19	0.631	0.201, 1.61	0.004	
